# New insights in Stevens Johnson syndrome/ toxic epidermal necrolysis syndrome

**DOI:** 10.1186/2045-7022-4-S3-P92

**Published:** 2014-07-18

**Authors:** Jan Schroeder, Maria Gloria Aversano, Antonella Citterio, Joseph Scibilia, Chiara Gamba, Corrado Mirone, Donatella Preziosi, Elide Anna Pastorello

**Affiliations:** 1 Niguarda Ca' Granda Hospital, Allergology and Immunology Unit, Italy; 2 Niguarda Ca' Granda Hospital, Burn/ Intensive Care Department, Italy; 3 Papa Giovanni XXIII Hospital, GISED Research Center, Italy

## 

Stevens Johnson syndrome (SJS) and toxic epidermal necrolysis (TEN) are rare, life-threatening cutaneous severe adverse reactions (SCAR) most frequently induced by a drug hypersensitivity. The clinical manifestations seem to be related either to a particular group of drugs, such as antibacterial sulfonamides, anticonvulsant agents, allopurinol, non-steroidal anti-inflammatory drugs (NSAIDs), aminopenicillins and quinolones, as well as to the ethnic/genetic background of the affected individual. A relevant problem in these subjects is the selection of a potentially tolerated drug that is less likely to induce a relapse of the reaction on the basis of cross-reactivity. Aim of the present study was to analyse a population of 16 patients that were treated in the burn/intensive care department for a SJS/TEN reaction, and determine the drugs that were tolerated after the reaction with respect to the culprit substances. The diagnosis was performed on the basis of the clinical and histological features according to the RegiSCAR classification and the first step was withdrawal of the offending drug. Among the 16 cases, we found 5 cases of SJS, 8 cases of TEN, and 3 cases of SJS/TEN overlap. Of the 16, 4 patients were male and 12 were female, age 14-92 years (average 57.1 years; median 63.5 years). A total of 12 drugs were found as suspected causative agents. In 3 cases multiple drugs were suspected, whereas in 13 cases a single drug was implicated. Antimicrobials including amoxicillin, levofloxacin, trimethoprim-sulfamethoxazole, vancomycin (8/16; 50 %) were the most commonly associated agents followed by antiseizure drugs (4/16; 25 %), NSAIDs (3/16; 18.75 %), benzodiazepines (2/16; 12.5 %) and allopurinol (1/16; 6.25 %). The most common complications noted were secondary infections (15/16; 93.75 %) and septicemia (11/16; 68.75 %). Mortality rate was 18,75 % (3/16). Interestingly patients with reactions to quinolones tolerated glycopeptides and carbapenems. Also, a patient with reaction to amoxicillin tolerated carbapenems. One of the 4 patients with reaction to anticonvulsant tolerated levetiracetam, 4/4 benzodiazepines, and 1/4 haloperidol. A patient with suspected reaction to acetaminophen (amoxicillin was the other involved drug) tolerated nimesulide and carbapenems. A patient with reaction to oxcarbazepine tolerated diazepam. We conclude that a competent burn centre staff can guarantee the successful management of the patients.

